# Identification and Synthesis of a Male-Produced Pheromone for the Neotropical Root Weevil *Diaprepes abbreviatus*

**DOI:** 10.1007/s10886-012-0096-8

**Published:** 2012-03-21

**Authors:** Stephen L. Lapointe, Rocco T. Alessandro, Paul S. Robbins, Ashot Khrimian, Ales Svatos, Joseph C. Dickens, Fernando Otálora-Luna, Fatma Kaplan, Hans T. Alborn, Peter E. Teal

**Affiliations:** 1 Agriculture Research Service, United States Department of Agriculture, U. S. Horticultural Research Laboratory, 2001 South Rock Road, Fort Pierce, FL 34945 USA; 2 Agriculture Research Service, United States Department of Agriculture, Invasive Insect Biocontrol and Behavior Laboratory, Beltsville, MD 20705 USA; 3 Max Planck Institute of Chemical Ecology, Jena, Germany; 4 Centro de Estudios Interdisciplinarios de la Física (CEIF), Instituto Venezolano de Investigaciones Científicas (IVIC), Mérida, República Bolivariana de Venezuela; 5 Agriculture Research Service, United States Department of Agriculture, Center for Medical, Agricultural, and Veterinary Entomology, Gainesville, FL 32608 USA

**Keywords:** Citrus root weevil, Pheromone, GC-EAD, Methyl (*E*)-3-(2-hydroxyethyl)-4-methyl-2-pentenoate, Coleoptera, Curculionidae, Crop pest

## Abstract

An unsaturated hydroxy-ester pheromone was isolated from the headspace and feces of male *Diaprepes abbreviatus*, identified, and synthesized. The pheromone, methyl (*E*)-3-(2-hydroxyethyl)-4-methyl-2-pentenoate, was discovered by gas chromatography-coupled electroantennogram detection (GC-EAD), and identified by gas chromatography–mass spectrometry (GC-MS) and nuclear magnetic resonance spectroscopy (NMR). The synthesis yielded an 86:14 mixture of methyl (*E*)-3-(2-hydroxyethyl)-4-methyl-2-pentenoate (active) and methyl (*Z*)-3-(2-hydroxyethyl)-4-methyl-2-pentenoate (inactive), along with a lactone breakdown product. The activity of the synthetic *E-*isomer was confirmed by GC-EAD, GC-MS, NMR, and bioassays. No antennal response was observed to the *Z-*isomer or the lactone. In a two-choice olfactometer bioassay, female *D. abbreviatus* moved upwind towards the synthetic pheromone or natural pheromone more often compared with clean air. Males showed no clear preference for the synthetic pheromone. This pheromone, alone or in combination with plant volatiles, may play a role in the location of males by female *D. abbreviatus*.

**Electronic supplementary material:**

The online version of this article (doi:10.1007/s10886-012-0096-8) contains supplementary material, which is available to authorized users.

## Introduction

The root weevil, *Diaprepes abbreviatus* (L.) (Curculionidae: Entiminae) (Marvaldi et al., 2002), is a major pest of citrus in the Caribbean and Florida. Prior to the 1960's, *D. abbreviatus* was reported only in the Caribbean; the multiple phenotypic populations that occur in Puerto Rico suggest this as its center of origin (Lapointe, 2004). Since discovery of *D. abbreviatus* near Apopka, Florida in 1964, this weevil has spread to Louisiana, Texas, and California leaving no geographic or climatic barrier to its movement south to Mexico, Mesoamerica, and South America (Lapointe et al., 2007). *Diaprepes abbreviatus* is typical of Entiminae in that the adults do not use the rostrum to create a niche for egg laying, and larvae feed externally on roots (Marvaldi et al., 2002). Adults feed and oviposit on foliage of a wide range of host plants (Simpson et al., 1996). Neonate larvae fall to the ground and burrow into the soil where they feed on progressively larger roots as they grow. Larvae pupate in the soil and emerge as adults throughout the year.

Within the superfamily Curculionoidea, the majority of known pheromones are long-range, male-produced aggregation pheromones (Seybold and Vanderwel, 2003; Ambrogi et al., 2009). Aggregations of *D. abbreviatus* adults have been observed on so-called "party trees" (Wolcott, 1936). Schroeder (1981) suggested that a *D. abbreviatus* male-produced pheromone attracted females, and a female-produced pheromone attracted males. Jones and Schroeder (1984) demonstrated that a male-produced pheromone in the feces attracted both sexes of *D. abbreviatus*, and there may be a pheromone responsible for arrestment behavior (Lapointe and Hall, 2009). Otálora-Luna et al. (2009) identified plant volatiles from citrus leaves that elicited antennal responses in *D. abbreviatus.* Such kairomones may act in concert with a pheromone to attract conspecifics to a suitable food source (Dickens et al., 1990).

We report here the isolation from headspace and feces of males, identification, and synthesis of a pheromone that attracts *D. abbreviatus* females.

## Methods and Materials

### Insects

Adult weevils were obtained from a laboratory colony maintained at the U. S. Horticultural Research Laboratory, Ft. Pierce, FL, USA, supplemented annually with field-collected adults. Neonate larvae were placed on artificial diet (product no. F1675, Bio-Serv, Inc., Frenchtown, NJ, USA) and reared as described by Lapointe et al. (2008). Adult males and females were held in separate 60 x 60 x 60 cm mesh cages with water-saturated dental wicks, and fed young citrus leaves (*Citrus macrophylla* Wester) until use in aerations or bioassays. Groups of 10–20 adults 4–6 wk-old were held without food for 24 h prior to behavioral assays. After the assay, adults were returned to their respective cages and provided food and water. Individual unmated adults were used in tests no more than once per week over a period of ~3 mo. Cohorts of known age were caged separately.

### Gas Chromatography-Electroantennogram Detection (GC-EAD)

The GC-EAD system consisted of an Agilent 7890A GC equipped with a split/splitless injector, an HP-1 capillary column (30 m x 0.32 mm x 0.25 μm, Agilent Technologies, Inc., Santa Clara, CA, USA), a post column glass Y-tube (Supelco, Bellefonte, PA, USA) splitter dividing the effluent in a 1:1 ratio between the flame ionization detector (FID) and heated EAD transfer line (200°C). Lengths of deactivated column (0.32 mm ID) were used to carry the effluent to the FID and EAD port after the split. The heated transfer line emptied into to a charcoal-filtered, humidified air stream (200 ml/min at 30 cm/sec) that carried the effluent over an antennal preparation. The air stream was directed past a probe attached to a type PRG-2 amplifier (Universal EAG probe, Syntech, Hilversum, The Netherlands). Signals from the amplifier and the FID were conditioned using a Syntech IDAC-2 interface. EAG and FID signals acquired from the IDAC-2 were displayed and stored on a computer running the GC-EAD 2011 software program (Syntech). An adult weevil antenna was mounted between the leads of the Universal EAG probe. The antennal preparation was made by plucking the antenna from the insect (grasping it firmly at the base of the antenna near the head with fine forceps) and placing it between two metal electrodes on the probe, to which small amounts of salt-free electrode gel (Spectra 360, Parker Laboratories, Fairfield, NJ, USA) had been applied. At the start of GC-EAD runs, the GC oven temperature was held at 35°C for 3 min, increased to 260°C at 15°C/min with a 10 min final hold. Injector and FID temperatures were 220°C and 300°C, respectively. Splitless injection was used with helium as the carrier gas at a flow rate of 2.3 ml/min.

### Analytical Methods

Coupled GC-mass spectrometry (GC-MS) was conducted using instruments operated in the electron impact (EI) and chemical ionization (CI) modes. EI spectra were obtained using an Agilent 5973 MS interfaced to a 6890 GC equipped with a cool on-column injector. The injector was fitted with a 10 cm length of 0.5 mm id deactivated fused silica tubing connected to 1 m (0. 25 mm id) length of deactivated fused silica tubing as a retention gap. The retention gap was connected to a 30 m x 0.25 mm id with 0.25 μm coating thickness DB5MS® analytical column. The injector and oven temperature were programmed from 30°C for 5 min to 225°C at 10°C/min. Spectra were obtained between 60–300 amu. Chemical ionization spectra were obtained using an Agilent 5975 MS interfaced to a 7890 GC. The GC was equipped with a cool on-column injector fitted with retention gaps as above. The analytical column used was a 30 m x 0.25 mm id, 0.25 μm coating thickness DB1MS®. The GC was operated using the same program as for EI spectra, and the CI spectra were obtained by scanning from m/z 60–300 using isobutane as reagent gas.

### Collection and Purification of Volatiles

Groups of 20 to 30 male and female *D. abbreviatus*, held separately, were placed in separate glass aeration chambers without plant material, and provided with a continuous flow (500 ml/min) of filtered, humidified air for 24 h at 27°C in an environmental chamber (12:12 h L:D). Volatiles were collected on Super Q filters (Alltech Deerfield, IL, USA) connected to the exit port of the aeration chambers. After collection, the filters were eluted with 500 μl of CH_2_Cl_2_. The accumulated feces in the aeration chambers at the end of the 24-h collection period were collected by washing the chambers with a minimum amount of CH_2_Cl_2_; the resulting extract was filtered and concentrated under nitrogen.

As an initial purification, CH_2_Cl_2_ eluates (200 μl) from the Super Q filters and washes of the aeration chambers were passed through Supelclean LC-SI solid phase extraction (SPE) columns containing 200 mg of packing (Supelco, Bellefont, PA) previously conditioned with 15 ml of CH_2_Cl_2_. The CH_2_Cl_2_ eluates plus a filter wash of 2 ml of CH_2_Cl_2_ were saved to check for the presence of the EAD-active compound. The SPE column was eluted with 2 ml each of pentane containing 15% ethyl acetate (EtOAc), 30% ETOAc, and 50% ETOAc. The three SPE column fractions, and the saved CH_2_Cl_2_ eluates, were analyzed by EI GC-MS for the presence of the EAD-active compound suspected to have a molecular weight of 172. Only the 15% EtOAc fraction contained the compound of interest. This fraction was concentrated to ca. 100 μl under a fine stream of N_2_ and subjected to fractionation by preparative GC. Initial fractionation was accomplished using an Agilent 6890 GC® with cool-on-column injector fitted with a 20 cm length of deactivated fused silica attached to a 30 m x 0.53 mm id (0.5 μm film thickness) DB1 column. The preparative column was split using a “Y” capillary connector between equal lengths of 0.1 mm id and 0.25 mm id lengths of deactivated fused silica column. The effluent from the 0.1 mm id column (ca. 13.8%) went to the FID, while the 0.25 mm id column (ca 86.2%) exited the wall of the GC and into the heated block (200°C) of a Brownlee-Silverstein collector (Brownlee and Silverstein, 1968). Ten μl samples were injected onto the column at an initial temperature of 30°C, and after 2 min the oven temperature was increased to a final temperature of 225°C at 10°C/min. The fractions were collected in a 30-cm liquid nitrogen-cooled glass capillary (Brownlee and Silverstein, 1968), and were recovered by washing the capillaries with 3 25-μl aliquots of CH_2_Cl_2_. Fractions were analyzed by GC-MS for the presence of compounds having *m/z* 154, 142, and 140 ions, all of which eluted within 0.01 min of each other. These fractions were combined, concentrated, and re-fractionated using a DB35 column (30 m x 0.53 mm id, 0.5 μm film thickness) as above. The fractions from this separation were eluted from capillaries using deuterated chloroform (99.96% CDCl_3_, Cambridge Isotope Laboratories, Inc.), analyzed by GC-MS, and the fractions containing the compound having the 172 MW were combined, concentrated under N_2_, and submitted for NMR analysis.

### NMR Analysis

One and two-dimensional NMR spectroscopy, including double-quantum filtered correlation spectroscopy (dqCOSY), heteronuclear single-quantum coherence (HSQC), heteronuclear multiple-bond correlation (HMBC), and Nuclear Overhauser Enhancement Spectroscopy (NOESY) were used to characterize the EAD-active compound. NMR spectra of the natural product were acquired at 27°C using a 5-mm TXI cryoprobe and a Bruker Avance II 600 console (600 MHz for 1H, 151 MHz for 13 C). The combined fractions containing the EAD-active compound (MW 172) were dissolved in ~150 μl CDCl_3_ and placed in a 2.5 mm NMR tube (Norell). Residual chloroform (CHCl_3_) was used to reference chemical shifts to δ(CHCl_3_) = 7.26 ppm for 1H and δ(CHCl_3_) = 77.36 ppm for 13 C (Gottlieb et al., 1997). Bruker Topspin 2.0 and Mestrelab MNova NMR (Mestrelab Research SL) software packages were used to process NMR spectra. ^1^H NMR spectra of synthetic materials, including a NOE difference spectrum for **1** *E*, were obtained on a Bruker AVIII-600 MHz spectrometer.

### Synthesis of the EAD-Active and Related Compounds

**Fig. 1:**
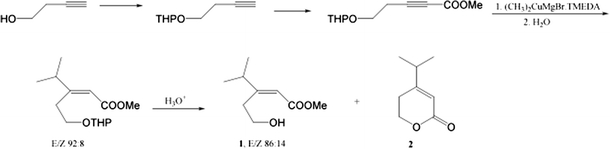
Synthetic route for methyl (*E*)-3-(2-hydroxyethyl)-4-methyl-2-pentenoate (**1**), and related compounds. See text for details

(Fig. 1)*.* Unless otherwise specified, all reagents and solvents were purchased from Aldrich Chemical Co. (Milwaukee, WI, USA). THP protection of 3-butyn-1-ol was conducted following a procedure of Rama Rao et al. (1986). A solution of 3-butyn-1-ol (14.0 g, 0.2 mol) in CH_2_Cl_2_ (75 ml, dried by distillation from CaH_2_) was placed in a flask under N_2_. The mixture was cooled to 0°C, and pyridine *p*-toluenesulfonate (0.28 g) was added. 3,4-Dihydro-2H-pyran (20.1 ml) was added via a dropping funnel maintaining the temperature between 0 and 5°C. The mixture was stirred at this temperature for 1 h, then warmed to 25°C, and stirred for an additional 2 h. TLC (silica gel, hexanes/ethyl acetate, 3:1) showed that the reaction was complete. The reaction mixture was taken into cold water (~ 70 ml), and transferred to a separatory funnel. The layers were separated, and the aqueous phase was extracted with CH_2_Cl_2_. Combined organic extracts were washed with NaHCO_3_ (aq), brine, and dried with Na_2_SO_4_ (anh). Distillation produced 23 g of THPO-protected 3-butyn-1-ol with bp 50-55°C/4 mm Hg. GC-MS (*m/z*, relative intensity): 153 (2, M^+^-1), 99 (9), 85 (100), 79 (9), 67 (20), 53 (42), 41 (33), a mass spectrum matching that presented in the NIST MS library for this compound.

A solution of the THPO-ether of 3-butyn-1-ol (2.88 g, 18.7 mmol) in THF (40 ml, dried with sodium benzophenone ketyl) was placed under N_2_ into a four-neck flask and cooled to −75°C. Butyl lithium (18.7 mmol, 9.4 ml of 2.0 M in hexanes) was added slowly via a dropping funnel at −75°C. The mixture was stirred at this temperature for 30 min, and then methyl chloroformate (ClCOOMe, 1.4 ml, 18.7 mmol) was added. The resulting mixture was stirred at −75°C for 30 min, then slowly warmed to 25°C in ~ 2 h and poured into a cold saturated NH_4_Cl solution. The organic material was extracted with hexanes/ether, 1:1. The combined extracts were washed with brine and dried. After removal of the solvent on a rotary evaporator, the remainder was flash chromatographed on silica gel with hexanes/ethyl acetate, 4:1, to isolate methyl 5-(tetrahydro-2H-pyran-2-yloxy)-2-pentynoate (2.5 g, 65%). GC-MS (*m/z*, relative intensity): 211 (1, M^+^-1), 157 (4), 153 (3), 142 (4), 125 (18), 113 (9), 109 (11), 85 (100), 79 (41), 67 (18), 55 (16), 41 (26). ^1^H NMR (CDCl_3_): 1.50-1.64 (m, 4H), 1.72 (m, 1H), 1.83 (m, 1H), 2.66 (t, *J* = 6.6 Hz, 2H, H-4), 3.53 (m, 1H, H-5A), 3.62 (dt, *J* = 9.8, 7.5 Hz, 1H, H-5B). 3.77 (s, 3H, OCH_3_), 3.87 (m, 2H), 4.65 (br t, *J* = 4.0 Hz, 1H, OCHO). ^1^H NMR data were in agreement with data obtained for this compound in CCl_4_ (Rama Rao et al., 1986).

Freshly purchased copper iodide (CuI, 1.97 g, 12.2 mmol) was placed under N_2_ in a four-neck flask. Dry tetrahydrofuran (THF) (35 ml) was added, followed by N,N,N’,N’-tetramethylethylenediamine (2.76 ml). The mixture was stirred at room temperature until a green-yellow solution was obtained and then cooled to −70°C, upon which a green suspension was formed. Isopropylmagnesium chloride solution (12.3 mmol; 6.1 ml of 2.0 M in THF) was added slowly, whereupon a green suspension became colorless then turned brown. The mixture was stirred at −70°C for 1 h, then methyl 5-(tetrahydro-2H-pyran-2-yloxy)-2-pentynoate (1.3 g, 6.1 mmol) dissolved in dry THF (5–10 ml) was added. The resultant mixture was stirred at −70°C for 3 h, and quickly poured into an ice-cold mixture of saturated NH_4_Cl and hexanes/ether, 5:1. The organic layer was separated, and the aqueous layer was extracted with hexanes/ether, 5:1. The combined organic extracts were washed thoroughly with saturated NH_4_Cl solution until no blue color was seen. The organic extract was dried and concentrated. Flash chromatography with hexanes/ethyl acetate, 5:1, afforded methyl 4-methyl-3-[2-(tetrahydro-2H-pyran-2-yloxy)ethyl]-2-pentenoate (1.3 g, 85%) as a 92:8 mixture of *E-* and *Z*-isomers as judged from GC-MS analysis. GC-MS (*E*-isomer, *m/z*, relative intensity): 172 (2), 171 (3), 155 (32), 142 (8), 141 (6), 139 (6), 95 (17), 85 (100), 67 (16), 57 (10), 55 (11), 43 (11), 41 (16). GC-MS (*Z*-isomer, *m/z*, relative intensity): 172 (4), 155 (3), 154 (8), 142 (8), 141 (2), 139 (4), 123 (6), 95 (23), 85 (100), 67 (17), 57 (10), 55 (12), 43 (11), 41 (17). ^1^H NMR (400 MHz, C_6_D_6_, δ): 0.86 (d, *J* = 8.0 Hz, *E*-isomer), 0.86 (d, *J* = 8.0 Hz, *Z*-isomer), 1,20-1.42 (m, 4H), 1.55-1.62 (m, 2H), 1.70-1.82 (m, 1H), 2.18-2.31 (m, 1H), 3.02-3.18 (m, 2H), 3.41 (s, OCH_3_), 3.67-3.73 (m, 1H), 3.81-3.88 (m, 1H), 4.04-4.11 (m, 1H), 4.39 (septet, *J* = 8.0 Hz, H-4, *Z*-isomer), 4.52 (t, *J* = 4.0 Hz, OCHO, *Z*-isomer), 4.66 (t, *J* = 4.0 Hz, OCHO, *E*-isomer), 5.83 (br. s, H-2, *E*-isomer), 5.86 (br. s, H-2, *Z*-isomer). ^13^ C NMR (101 MHz, C_6_D_6_, δ, *E*-isomer): 20.0, 21.7 (two carbons), 26.3, 31.4, 32.9, 37.3, 50.9, 61.9, 67.3, 98.8, 115.1, 167.2, 167.8.

Methyl 4-methyl-3-[2-(tetrahydro-2H-pyran-2-yloxy)ethyl]-2-pentenoate (256 mg, 1 mmol) was stirred with *p*-toluenesulfonic acid hydrate (9 mg, 0.047 mmol) in a THF-H_2_O solution (8 + 2 ml) at 55-60°C for ~ 1 h, or until TLC analysis (SiO_2_ plates; hexanes/ethyl acetate/MeOH, 16:6:1; visualization with KMnO_4_ solution) showed very little starting ester present. The mixture was cooled to room temperature, treated with 50 μl 1 N NaOH, and concentrated to remove most of the THF. The mixture was extracted with ether/hexanes, 1:1, and the organic extract was dried with Na_2_SO_4_ (anh). After evaporation of the solvent, the remainder was flash chromatographed with hexanes/ethyl acetate/MeOH, 16:6:1. Two fractions were obtained: a) starting THPO-ester, 16 mg; and b) a mixture of ester **1** and lactone **2** (Fig. 1). The second fraction was chromatographed again with hexanes/ethyl acetate/MeOH, 16:6:1 to furnish **1** (*E/Z* 86:14, 90 mg, 58%) in the less polar fraction. ^1^H NMR (600 MHz, C_6_D_6_, δ): 0.79 (d, *J* = 6.6 Hz, (CH_3_)_2_, *E*), 0.91 (d, *J* = 6.6 Hz, (CH_3_)_2_, *Z*), 2.01-2.08 (m, H-4 *E*, CH_2_C = C, *Z*), 2.46 (t, *J* = 5.4 Hz, OH, *E*), 2.76 (t, *J* = 6.6 Hz, CH_2_C = C, *E*), 3.34 (s, OCH_3_, *E*), 3.36-3.38 (m, CH_2_OH, *Z*), 3.41 (s, OCH_3_, *Z*), 3.70 (q, *J* = 5.4 Hz, CH_2_OH, *E*), 4.32 (septet, H-4, *Z*), 5.71 (br. s, H-2, *Z*), 5.80 (br. s, H-2, *E*). ^13^ C NMR (151 MHz, C_6_D_6_, *E*-isomer): 21.7 (two carbons), 35.6, 36.7, 51.1, 62.5, 115.6, 167.7, 168.7; *Z*-isomer: 20.9 (two carbons), 29.8, 35.1, 50.8, 61.6, 116.0, 165.7, 166.8. Lactone **2** (10 mg) was recovered from the more polar (second) fraction. GC-MS (*m/z*, relative intensity): 140 (M^+^, 16), 125 (7), 110 (15), 97 (19), 96 (59), 95 (96), 82 (24), 81 (100), 67 (73), 55 (17), 41 (40). ^1^H NMR (400 MHz, C_6_D_6_, δ): 0.57 (d, *J* = 6.6 Hz, (CH_3_)_2_), 1.37 (br. t, *J* = 6.5 Hz, CH_2_C=), 1.70 (septet, *J* = 6.6 Hz, CH(CH_3_)_2_), 3.61 (t, *J* = 6.5 Hz, CH_2_O), 5.67 (d, *J* = 1.0 Hz, CHC=). NMR data are in agreement with those obtained for this compound in CDCl_3_ (D’Annibale et al., 2007).

NMR signals of the synthetic compound in CDCl_3_ were: ^1^H (400 MHz): 1.12 (d, *J* = 6.8 Hz, (CH_3_)_2_, *E*), 2.44 (septet,*J* = 6.5 Hz, CH(CH_3_)_2_), 2.87 (t, *J* = 6.4 Hz, CH_2_C=), 3.73 (s, OCH_3_), 3.81 (br. q, 5.2 Hz, CH_2_OH), 5.85 (br. s, CH=). ^13^ C (101 MHz): 21.5 (two carbons), 34.7, 36.2, 51.3, 62.0, 115.3, 166.8, 168.7.

### Confirmation of EAD-Activity and Behavioral Response to Synthetic **1**E

The synthetic methyl (*E*)-3-(2-hydroxyethyl)-4-methyl-2-pentenoate was diluted with hexane to approximately 100 ng/μl, and 1 μl of sample were injected on the GC-EAD system described above. A 1-μl injection of 50 ng of linalool and 100 ng/μl of the synthetic methyl (*E*)-3-(2-hydroxyethyl)-4-methyl-2-pentenoate in hexane was used to determine retention times and test antennal responses. Linalool was determined previously to elicit a consistent antennal response, and was co-injected with methyl (*E*)-3-(2-hydroxyethyl)-4-methyl-2-pentenoate to confirm that antennae were viable. Antennae from male and female *D. abbreviatus* were tested.

Behavioral response to the pheromone was tested in an olfactometer (Model 4 C, ARS, Inc., Micanopy, FL USA). Individual weevils (starved for 24 h) were placed in a glass inlet, allowed to walk upwards into the center of an arena with a balanced, filtered, and humidified airflow from two arms oriented at 180° to each other and outfitted with glass reservoirs containing an odor source or blank. The assays were conducted in the dark between 9 am and 2 pm because of a strong phototropic response in this insect (Lapointe and Hall, 2009). Each run was terminated when the weevil moved into one of the glass receptacles or after 15 min. Weevil position was scored as no-response (remaining in the inlet), no-choice (moving to, but remaining in the central arena), or choosing one of the two arms. Responses of unmated 4-6-wk-old males and females were recorded to various odor sources: fresh young citrus leaves (flush), flush fed upon for 24 h by male *D. abbreviatus*, 30 μg of methyl (*E*)-3-(2-hydroxyethyl)-4-methyl-2-pentenoate in 10:1 hexanes:ethyl acetate pipetted onto a glass slide, and a clean glass slide. All glass components of the olfactometer were washed thoroughly between runs with warm soap and water, rinsed with methanol, and air-dried. Between replications of a given treatment, the arms of the olfactometer used for that treatment were switched to control for bias in the apparatus. The number of weevils choosing a treatment arm was compared with the control arm (clean air) by the *G*-test (Sokal and Rohlf, 1994).

## Results

**Fig. 2:**
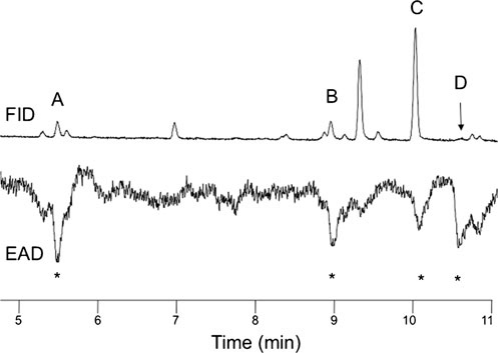
Simultaneous gas chromatogram (FID) and electroantennogram detection (EAD) of a male *Diaprepes abbreviatus* antenna responding to a hexane extract of headspace volatiles collected from adult males and citrus leaves. Compounds A - C correspond to the plant volatiles: linalool, geraniol and citral, respectively; compound D is male-derived. Asterisks indicate consistent antennal responses

**Fig. 3:**
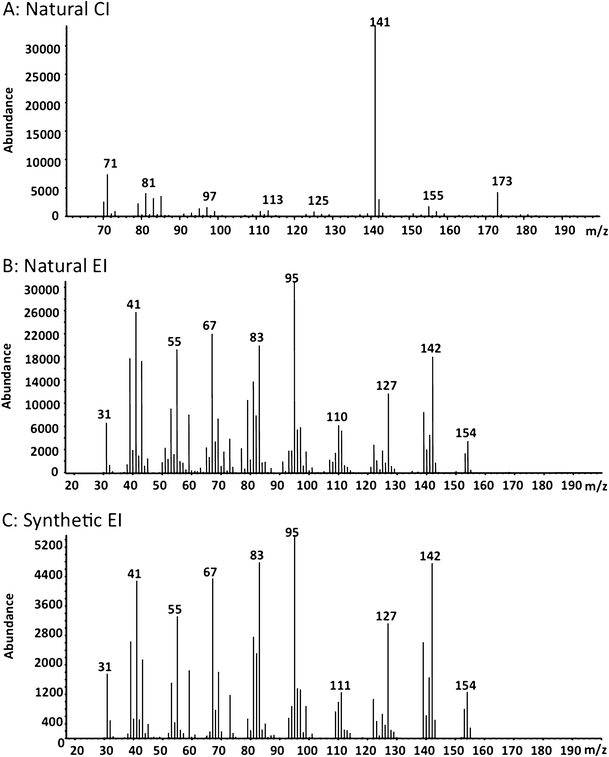
Chemical ionization **a** and electron impact **b** mass spectra of the EAD-active compound from an aeration extract of *Diaprepes abbreviatus* males, and the EI mass spectrum of synthetic methyl (*E*)-3-(2-hydroxyethyl)-4-methyl-2-pentenoate **c**. The CI spectrum shows the M + 1 ion at m/z 173, thus supporting a molecular weight of 172 Daltons

One compound from aerations of male weevils consistently elicited antennal responses from both male and female *D. abbreviatus* in GC-EAD experiments (Fig. 2). The amount of this compound in 24-h aerations of ten *D. abbreviatus* males was sufficient to elicit responses from the antennae of males and females, but the compound was barely detectable by GC. The same compound also was recovered in CH_2_Cl_2_ by washing the frass of males from glass aeration jars. Combining multiple aerations of males provided sufficient material to obtain CI and EI mass spectra (Fig. 3a, b). Initial GC analyses indicated the presence of several compounds eluting in the area of the EAD-active compound (Fig. 2). EI GC-MS analyses indicated that several of these, including the EAD-active compound, had an *m/z* 154 ion and fragmentation patterns corresponding to monoterpene alcohols. Although the EI mass spectrum of the EAD-active compound showed an *m/z* 154 ion (Fig. 3b), the presence of an *m/z* 142 ion indicated that *m/z* 154 might not be the molecular ion; the latter possibility was verified by SIM analysis showing *m/z* 140, 142, and 154 ions maximized within ±0.01 min of each other. CI-MS analysis (Fig. 3a) exhibited a clear M + 1 ion at *m/z* 173, thus supporting a molecular weight of 172 Daltons for the EAD-active compound. In addition, the CI analysis also exhibited a base peak at *m/z* 141 (M + 1-CH_3_OH), and a prominent *m/z* 155 ion (M + 1-18); thus, the *m/z* 154 ion in the EI spectrum is due to loss of H_2_O. The CI did not directly indicate the origin of the *m/z* 142 fragment in the EI spectra; however, the loss of 30 amu (*m/z* 172–142) suggested a long range proton transfer to a carbonyl group followed by a neutral loss of CH_2_ = O, which is consistent with a hydrocarbon chain having a terminal alcohol as well as a carbonyl. This interpretation also was congruent with a methyl ester as suggested by the (M + 1-CH_3_OH) loss in the CI spectrum. A characteristic neutral loss of 32 amu also was seen in the EI spectra (*m/z* 142*–*110), thereby supporting the structure of a methyl ester. Furthermore, the neutral loss of 15 amu (*m/z* 142–127 as well as *m/z* 110–95) suggested the presence of a methyl branch. Thus, the fragmentation patterns indicated a formula of C_9_H_16_O_3_ with 2 degrees of unsaturation, the presence of a methyl ester, and at least one methyl branch, and a terminal C-OH. Further aerations of groups of up to 30 males, and fractionations by preparative GC using both polar and nonpolar capillary columns, resulted in collection of a sufficient amount of the pheromone for NMR analysis (Table 1). These experiments enabled the structure assignment for the EAD-active peak as methyl (*E*)-3-(2-hydroxyethyl)-4-methyl-2-pentenoate (**1** *E)*. The NMR analysis also revealed an antennally inactive isomer (methyl (*Z*)-3-(2-hydroxyethyl)-4-methyl-2-pentenoate), and a related lactone **2** (Table 2) that apparently originated from partial isomerization and lactonization of the putative pheromone catalyzed by CDCl_3_.

**Table 1:**
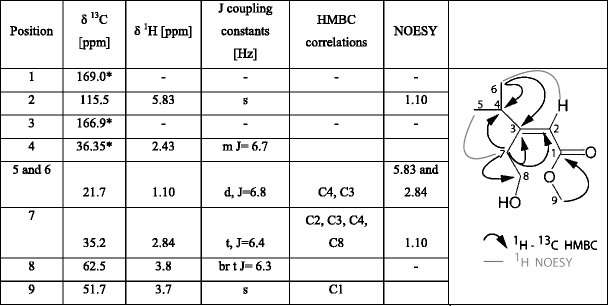
^1^H (600 MHz), ^13^ C (151 MHz), HMBC and NOESY NMR spectroscopic data for the putative pheromone of *Diaprepes abbreviatus* in CDCl_3_. Chemical shifts referenced to δ(CHCl_3_) = 7.26 ppm for ^1^H and δ(CHCl_3_) = 77.36 ppm for ^13^ C. Coupling constants are given in Hertz [Hz]

*The ^13^ C chemical shifts are deduced from HMBC; others are deduced from HSQC. ^1^H chemical shifts are deduced from 1D ^1^H NMR

**Table 2:**
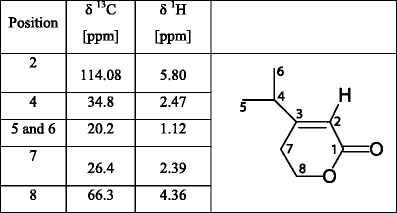
^1^H (600 MHz) and ^13^ C (151 MHz) spectroscopic data for the lactone degradation product present in aeration extracts of *Diaprepes abbreviatus* males. Only HSQC data are reported for the lactone. Chemical shifts referenced to δ(CHCl_3_) = 7.26 ppm for ^1^H and δ(CHCl_3_) = 77.36 ppm for ^13^ C. Numbering is the same as in Table 1

The structure of the synthetic compound was confirmed as identical with the natural EAD-active compound by mass spectral (Fig. 3c) and NMR analyses. As with the natural material, the synthetic material contained a pair of Z/E-isomers of methyl 3-(2-hydroxyethyl)-4-methyl-2-pentenoate, along with the lactone breakdown product. The NMR of the synthetic sample obtained in C_6_D_6_ showed two doublets at 0.79 and 0.91 ppm (corresponding to the doublets at 1.09 and 1.12 ppm in the isolated material) due to a pair of methyl groups from both stereoisomers split by a single proton on the adjacent carbon. Similarly, there was a pair of singlets at 3.34 and 3.41 ppm due to methyl groups from isomeric esters corresponding to the singlets at 3.73 and 3.71 ppm in the NMR of natural sample recorded in CDCl_3_. A NOE difference spectrum obtained by irradiating the resonance of the larger doublet at 0.79 ppm resulted in enhancement of the larger singlet from the olefinic proton at 5.80 ppm and vice versa. Therefore, the olefinic proton and the isopropyl group in the major stereoisomer are in *cis*-position, and this stereoisomer and the natural product have the *E*-configuration.

### Synthesis and Stability of the EAD-Active Compound

The synthesis of methyl (*E*)-3-(2-hydroxyethyl)-4-methyl-2-pentenoate **1** is depicted in Fig. 1. The chosen route employs stereoselective carbocupration of α,β-acetylenic esters that is exclusively *cis*-stereospecific when the reaction is conducted in THF at low temperatures (Corey and Katzenellenbogen, 1969; Bourque et al., 1999; Drew et al., 1999). However, a conjugate addition of a heterocuprate, formed in situ from isopropylmagnesium bromide and copper(I) iodide in the presence of N,N,N',N'-tetramethylethylenediamine (Crimmins et al., 1984), to the acetylenic ester (Fig. 1) proceeded with some loss of stereoselectivity resulting in a mixture of *E* and *Z* olefinic esters in a 92:8 ratio. During acid-catalyzed removal of the tetrahydropyranyl (THP) protecting group, the ratio of **1** *E* and **1** *Z* dropped to 86:14, and a considerable amount of lactone **2** was formed, thus indicating instability of the putative pheromone under acidic conditions. A noticeable cyclization of **1** *E* to **2** also occurred in the acidic CDCl_3_ used for recording NMR spectra. Using *t*-butyldimethylsilyl (TBDMS) protecting group (instead of THP) for the acetylenic alcohol provided a similar stereochemistry at the carbocupration (*E*/*Z* 93:7) step but, again, resulted in significant lactonization when the TBDMS group was removed with highly basic tetrabutylammonium fluoride. Lactonization also occurred when a purified sample of the synthetic (*E*)-3-(2-hydroxyethyl)-4-methyl-2-pentenoate was injected at 260°C into the split-splitless injection port of the GC- MS, resulting in a total ion chromatogram comprising **1** *E*:**1** *Z*:**2** in a ratio of 39:8:53; the same sample analyzed by cool-on-column GC-MS injection yielded an 88:9:3 mixture of **1** *E*:**1** *Z*:**2**. Because of its thermal instability, initial attempts to isolate the putative pheromone by preparative GC equipped with a conventional injection port failed. A purified sample of the synthetic compound could be stored indefinitely at 0-25°C in aprotic solvents, such as benzene, hexane, and ethyl acetate.

### Confirmation of EAD-Activity and Behavioral Response to Synthetic **1**E

**Fig. 4:**
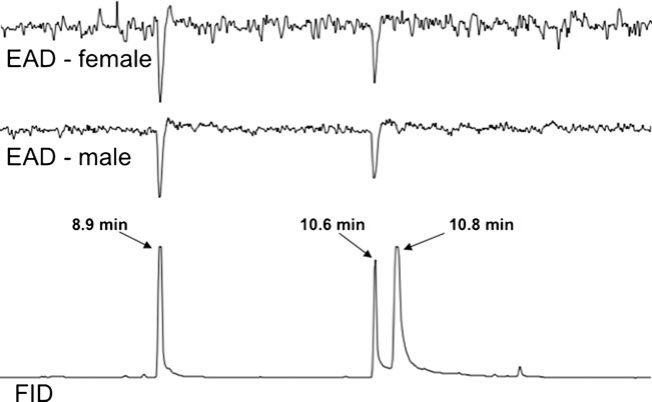
Simultaneous gas chromatogram (FID) and electroantennogram detection (EAD) of antennae from a female and a male *Diaprepes abbreviatus* responding to 50 ng of linalool (RT 8.9 min) and 100 ng of synthetic methyl (*E*)-3-(2-hydroxyethyl)-4-methyl-2-pentenoate (10.6 min) in hexane. The peak at 10.8 min corresponds to the lactone degradation product (**2)**

The antennae of both males and females responded to methyl (*E*)-3-(2-hydroxyethyl)-4-methyl-2-pentenoate, and neither responded to the *Z*-isomer or to the lactone (Fig. 4). In two-choice olfactometer tests, female weevils consistently moved upwind more often (α = 0.05, *G*-test) to the olfactometer arm containing either 30 μg of methyl (*E*)-3-(2-hydroxyethyl)-4-methyl-2-pentenoate (**1** *E*) or citrus leaves previously fed upon by males (MFUF) than to the arm with clean air (Table 3). Males did not show a clear preference for the **1** *E* or MFUF compared with clean air. Neither males nor females showed a preference when offered a choice between clean air and citrus leaves (flush). Results not reported here suggest that males may be attracted or repelled by the odor of other males due to factors we do not yet understand.

**Table 3:** Behavioral responses of *Diaprepes abbreviatus* adults in a two-choice olfactometer to citrus leaves fed upon by males (MFUF; a natural source of pheromone), and the synthetic EAD-active compound, methyl (*E*)-3-(2-hydroxyethyl)-4-methyl-2-pentenoate. Tests were conducted in the dark; adults were allowed 15 min to respond; results were scored as no response (weevils remained at the starting point), no choice (weevils moved into the arena but failed to move into one of the two olfactometer arms), or response by moving into one of the arms. Significance is reported for the two-tailed contrast between the number of weevils that chose A or B

	No. choosing					
	Sex	A	B	No choice	No response	Pr ≥ *G*
A: Air; B:30 μg methyl (*E*)-3-(2-hydroxyethyl)-4-methyl-2-pentenoate						
	Male	30	34	20	69	N.S.
	Female	17	48	17	71	< 0.001
A: Air; B: MFUF						
	Male	14	15	6	5	N.S.
	Female	6	20	13	13	0.009
A: Air, B: Air						
	Male	12	18	6	32	N.S.
	Female	6	9	13	36	N.S.
A: Air, B: Citrus leaves						
	Male	4	4	2	13	N.S.
	Female	4	3	7	7	N.S.

## Discussion

An attractant has been sought for *D. abbreviatus* for over 30 years. Our GC-EAD and GC-MS analyses of headspace volatiles and frass collected from male *D. abbreviatus* provided the initial evidence of a putative pheromone consisting of a single, novel compound. GC-MS and NMR analyses enabled us to determine the structure of the EAD-active compound as methyl (*E*)-3-(2-hydroxyethyl)-4-methyl-2-pentenoate (***1*** *E*). We then synthesized the putative pheromone compound, and showed that the synthetic compound elicited positive antennal and olfactometer responses verifying that this compound is an attractant pheromone for female *D. abbreviatus*. Whereas the pheromone **1** *E* is novel, its lactone degradation product **2** was described as a constituent of tobacco flavor (Pettersson et al., 1993), and synthesized (D’Annibale et al., 2007; Brichacek and Carlson, 2007). As with other curculionids (Ambrogi et al., 2009), the *D. abbreviatus* attractant pheromone is male-produced; however, in the bioassays conducted to date, we have demonstrated a significant attraction of conspecific females but not males. This represents the first progress towards development of semiochemical-based methods for managing *D. abbreviatus*, a serious pest of many crops and ornamental plants that is expanding its geographic range in the Neotropics and subtropics.

Schroeder (1981) conducted field tests suggesting that *D. abbreviatus* weevils were attracted to trees that had been infested overnight by conspecifics of the opposite sex, thereby implying the existence of two sex-specific aggregation pheromones. Subsequently, Beavers et al. (1982) conducted olfactometer bioassays showing *D. abbreviatus* attraction to the frass of the opposite sex, along with same-sex attraction. Both Jones and Schroeder (1984), and Schroeder and Beavers (1985) then conducted field trapping experiments demonstrating that extracts of male *D. abbreviatus* attracted both *D. abbreviatus* males and females; attraction to frass from males was also demonstrated by Harari and Landolt (1997). Lapointe and Hall (2009) found that males responded by arresting their movement on citrus leaves previously fed and defecated upon by males or females, thereby suggesting the presence of a female-produced pheromone. From these reports and our own data, we believe there are volatile compounds of insect origin, in addition to methyl (*E*)-3-(2-hydroxyethyl)-4-methyl-2-pentenoate, that influence the behavior of *D. abbreviatus*. Our data do not preclude the existence of a female-produced pheromone. The consistent suggestion that female frass also contains an attractant merits further study. Discovery of the male-produced pheromone in *D. abbreviatus* appears to be only the first step towards elucidating the chemical ecology of this highly polyphagous and damaging weevil.

Among the Entiminae (i.e., the broad-nosed weevils), the only one other pheromone known is the aggregation pheromone of *Sitona lineatus* (4-methyl-3,5-heptanedione) (Blight et al., 1984). Blight and Wadhams (1987) suggested that *S. lineatus* produces its aggregation pheromone in the spring, and that pheromonal activity is synergized by host plant volatiles, including (*Z*)-3-hexen-1-ol and linalool. These compounds, and others isolated from headspace over citrus leaves, consistently elicit antennal responses from *D. abbreviatus* (Otálora-Luna et al., 2009). It remains to be seen if these plant volatiles synergize or otherwise augment activity of the *D. abbreviatus* pheromone, and we do not understand the potential influence of rearing conditions, seasonality, age or mating status on production or response to methyl (*E*)-3-(2-hydroxyethyl)-4-methyl-2-pentenoate.

## Electronic supplementary material

Below is the link to the electronic supplementary material.

Esm 1 (JPEG 110 kb)

High resolution image (TIFF 2494 kb)
