# Supplementary figures and images for: Identification and Synthesis of a Male-Produced Pheromone for the Neotropical Root Weevil Diaprepes abbreviatus
Source: J Chem Ecol. 2012 Mar 21;38(4):408–17. doi: 10.1007/s10886-012-0096-8 (PMC3324679; doi:10.1007/s10886-012-0096-8)

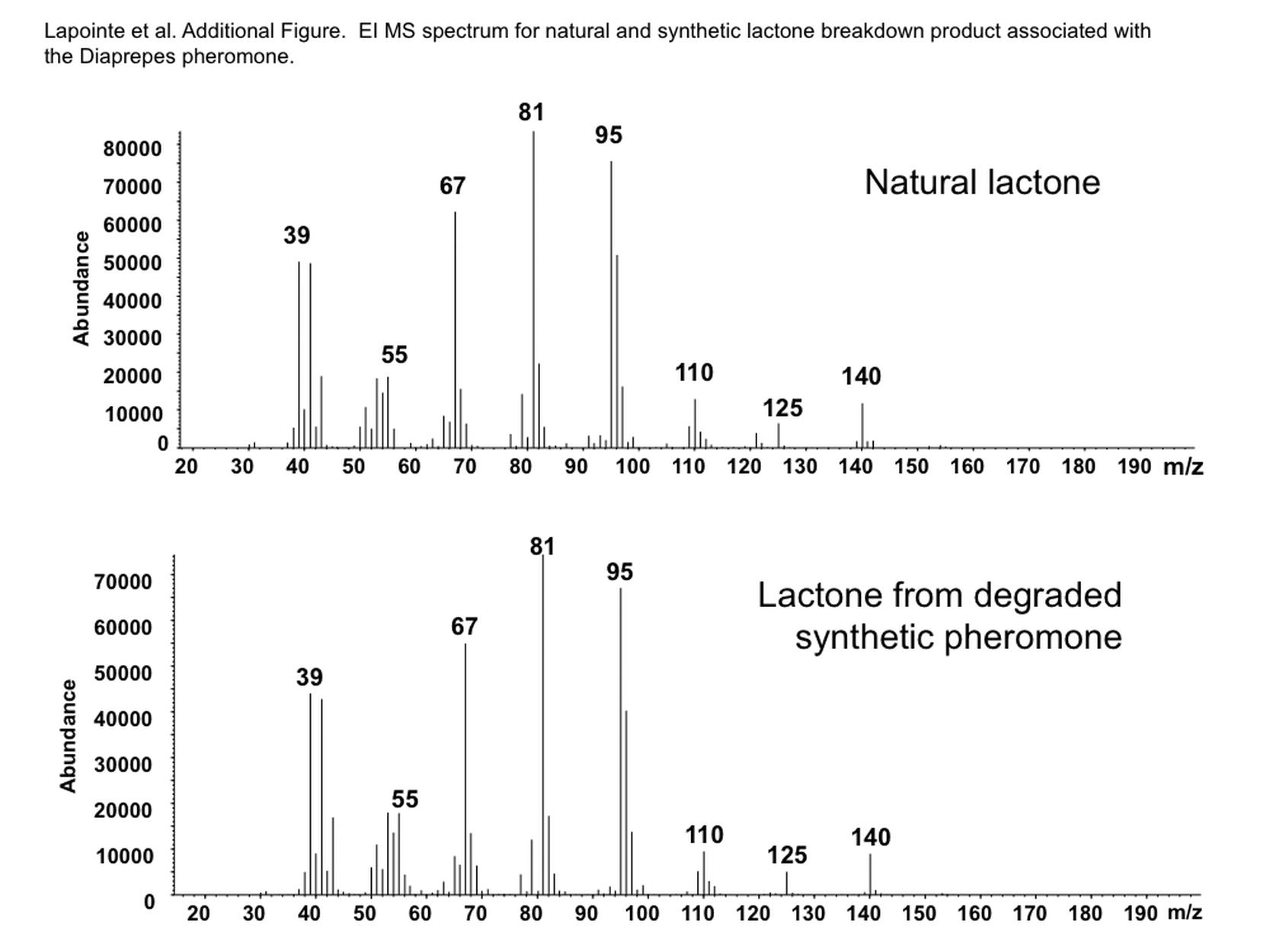

Supplement: Supplementary file 1 — (JPEG 110 kb) [file 10886_2012_96_Fig5_ESM.jpg]

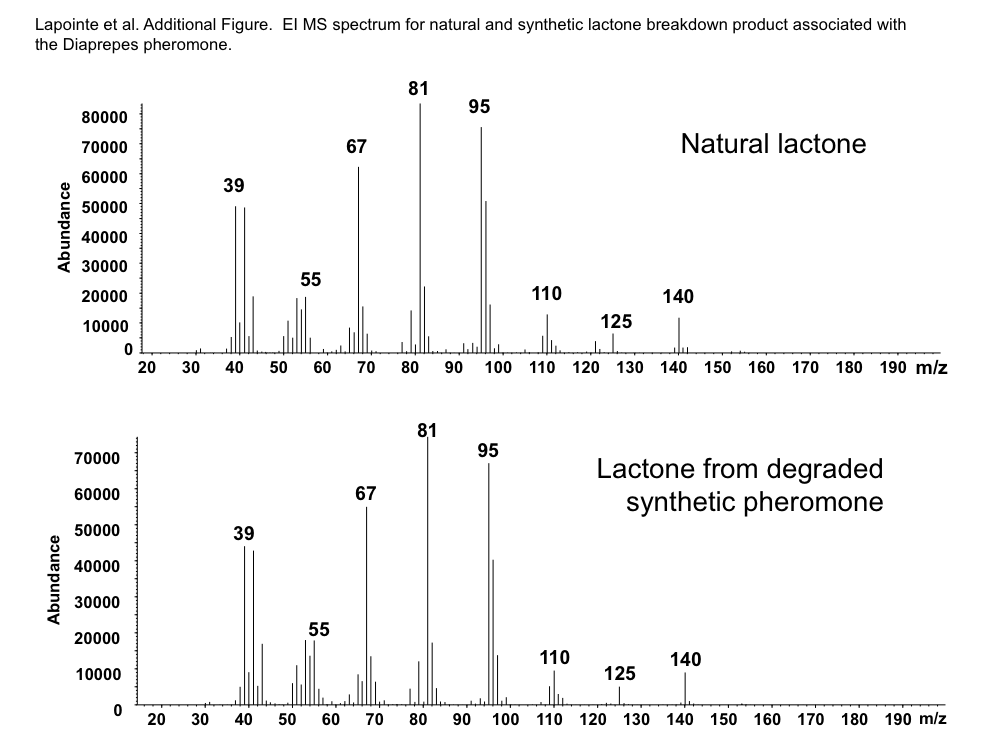

Supplement: Supplementary file 2 — High resolution image (TIFF 2494 kb) [file 10886_2012_96_MOESM1_ESM.tif]
